# Depth-specific fluctuations of gene expression and protein abundance modulate the photophysiology in the seagrass *Posidonia oceanica*

**DOI:** 10.1038/srep42890

**Published:** 2017-02-17

**Authors:** Gabriele Procaccini, Miriam Ruocco, Lázaro Marín-Guirao, Emanuela Dattolo, Christophe Brunet, Daniela D’Esposito, Chiara Lauritano, Silvia Mazzuca, Ilia Anna Serra, Letizia Bernardo, Amalia Piro, Sven Beer, Mats Björk, Martin Gullström, Pimchanok Buapet, Lina M. Rasmusson, Paulo Felisberto, Sylvie Gobert, John W. Runcie, João Silva, Irene Olivé, Monya M. Costa, Isabel Barrote, Rui Santos

**Affiliations:** 1 Stazione Zoologica Anton Dohrn, Naples, Italy; 2 Universitá della Calabria, Rende, Italy; 3 Tel Aviv University, Tel Aviv, Israel; 4 Stockholm University, Stockholm, Sweden; 5 Prince of Songkla University, Songkhla, Thailand; 6 LARSyS, University of Algarve, Faro, Portugal; 7 MARE Centre, University de Liege, Liege, Belgium; 8 University of Sydney, Sydney, Australia; 9 CCMAR, University of Algarve, Faro, Portugal

## Abstract

Here we present the results of a multiple organizational level analysis conceived to identify acclimative/adaptive strategies exhibited by the seagrass *Posidonia oceanica* to the daily fluctuations in the light environment, at contrasting depths. We assessed changes in photophysiological parameters, leaf respiration, pigments, and protein and mRNA expression levels. The results show that the diel oscillations of *P. oceanica* photophysiological and respiratory responses were related to transcripts and proteins expression of the genes involved in those processes and that there was a response asynchrony between shallow and deep plants probably caused by the strong differences in the light environment. The photochemical pathway of energy use was more effective in shallow plants due to higher light availability, but these plants needed more investment in photoprotection and photorepair, requiring higher translation and protein synthesis than deep plants. The genetic differentiation between deep and shallow stands suggests the existence of locally adapted genotypes to contrasting light environments. The depth-specific diel rhythms of photosynthetic and respiratory processes, from molecular to physiological levels, must be considered in the management and conservation of these key coastal ecosystems.

Seagrasses are marine angiosperms that have evolved to complete their whole life cycle submerged. They represent one of the most productive components of benthic coastal ecosystems, and in addition to being exposed to daily fluctuations in irradiance have adapted to large spatial (i.e. bathymetric) variations in both irradiance and spectral quality. In the Mediterranean Sea, the endemic seagrass *Posidonia oceanica* colonizes extensive portions of sea bottom along most of the coastline^1^. The extensive meadows formed by this species are among the most valuable marine ecosystems, fulfilling important ecosystem services from carbon sequestration to coastal protection and maintenance of fisheries^2^,^3^,^4^. Meadow can spread from the surface down to 45 meters depth, where distinct ramets (but possibly the same genets^5^,^6^) are exposed to a wide range of light conditions and different photoperiods, imposing specific adaptive responses. Such extensive *P. oceanica* meadows offer a valuable opportunity to explore how depth affects light responses and diel rhythms in seagrasses.

The amplitude of physiological changes of the species associated with depth-related variations in light is similar to the one shown by the congeneric *P. sinuosa*^7^ and can be considered low in relation to other seagrasses^8^,^9^. These changes include modifications in the plants’ photosynthetic and respiratory rates and in the ability of leaves to harvest light (e.g. refs 10,11) and involve long-lasting adjustments of key metabolic processes including carbon metabolism, stress defense and proteolysis. As a response to low-light conditions, regulatory changes of important functional groups of proteins have been reported, including down-regulation of the Ribulose-1,5-Bisphosphate Carboxylase/Oxygenase (RuBisCO) large subunit and modification of the PSI/PSII ratio^12^. Additionally, divergence in the level of expression of photoacclimation- and photoprotection-related genes (i.e. RuBisCO, ferredoxin, chlorophyll *a/b* binding proteins, zeaxanthin epoxidase), and antioxidant enzymes, has recently been observed in plants from contrasting depths^13^,^14^. High-light adaptation/acclimation in shallow stands include a decrease in the antenna size, and an enhancement of photoprotective mechanisms (i.e. the xanthophyll cycle), as well as an increase in the number of reaction centers^14^. *P. oceanica* did not show signs of stress response in deeper water^14^, suggesting that the plant is well adapted to low-light environments.

Marine macrophytes show circadian rhythms, since much of their biochemistry, physiology, and behavior is temporally organized with respect to the environmental oscillation of day and night^15^,^16^. Circadian rhythms control many functions in plants such as development and growth^17^. The correct matching of the endogenous clock with the day-night cycle directly enhances photosynthesis, increasing productivity and conferring fitness^18^.

Seagrass responses to daily changes in the light regime have been studied almost exclusively at the photophysiological level^19^,^20^,^21^,^22^,^23^,^24^,^25^,^26^. According to daily variation in irradiance levels, seagrasses, like their terrestrial counterparts, maintain a permanent and dynamic trade-off between photosynthetic efficiency and photoprotection through so-called dynamic photoinhibition^27^. It is not known whether and how this reversible regulation of photosynthesis is driven by internal circadian cycles, allowing the species to anticipate upcoming light patterns (i.e. seasonally or daily), or is regulated by environmental cues. Given the important role temporal rhythms can play in the functioning of seagrass meadows, it is of great importance to explore the basis of their genetic, biochemical and physiological attributes.

Here we present the results of a multidisciplinary study conceived to understand the acclimative and adaptive strategies of *P. oceanica* photophysiology to the daily fluctuations in the light environment, at contrasting depths. The study was performed in the *P. oceanica* meadow just outside the Marine Research Station of STARESO, Bay of Calvì (Corsica), and plants were collected during a daily cycle at −5 m and −20 m (cf. ref. 28). Irradiance, photophysiological parameters derived from chlorophyll *a* fluorescence measures, leaf respiratory rates, content of photoprotective xanthophyll pigments, leaf total protein and mRNA levels of selected genes and their relative expression, were evaluated.

The spatial genetic structure along the depth gradient was also assessed. Together with a companion study (in preparation), performed at the same time, which focuses on how plant photosynthetic production is conveyed to higher organizational levels of community and ecosystem production, we provide the first comprehensive, multilevel approach of the functional responses of a seagrass ecosystem to the daily irradiance cycle at different depths. Our results represent an important source of information on the adaptive strategies used by this species, in response to variations in light, and provide a better understanding of the overall functioning of *P. oceanica* meadows.

## Results

### Light and temperature

**Figure 1:**
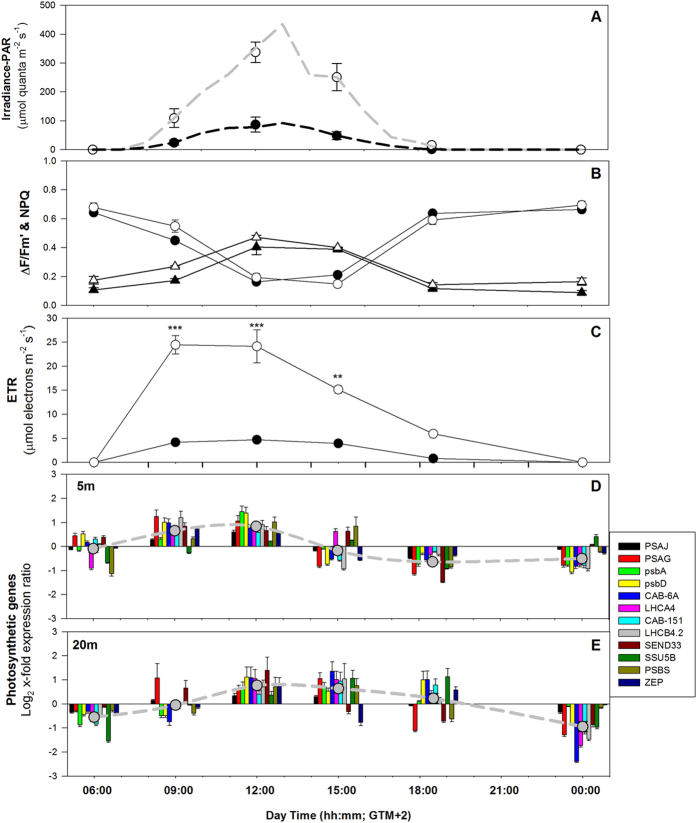
Daily cycle of PAR irradiance (**A**), quantum yield of PSII (circles in **B**), non-photochemical quenching downregulation (triangles in (**B**)) and electron transport rates (**C**) of shallow (white symbols) and deep (black symbols) *P. oceanica* plants; and daily variation of mRNA expression of photosynthesis-related genes at 5 m (**D**) and 20 m (**E**) depth. Relative expression levels at given time points are calculated over their daily average expression. Dashed lines represent the average expression level of all analyzed genes. Asterisks indicate significant differences among shallow and deep plants at a given sampling time. **p < 0.01; ***p < 0.001.

During the study period, dawn and dusk (as defined by PAR < 1 μmol m^−2^ s^−1^) at the shallow (−5 m) portion of the STARESO *P. oceanica* meadow occurred at approximately 07:25 and 19:00 GMT + 2, respectively. At −20 m the daily photoperiod was shortened by 25–30 min. Maximum PAR values at both depths were recorded around 13:00 with averaged values of 430 and 92 μmol m^−2^ s^−1^ at 5 and 20 m depth, respectively (Fig. 1A). During the first sampling after sunrise (i.e. at 09:00), available light levels were 109 ± 32 μmol m^−2^ s^−1^ at 5 m and 24 ± 9 μmol m^−2^ s^−1^ at 20 m depth. The integrated daily photosynthetic photon flux density at −20 m was on average 24% of the corresponding value at −5 m. Seawater temperature was similar at the two depths, with daily averaged values of 20.4 ± 0.1 °C and 20.0 ± 0.1 °C for shallow and deep meadow portions, respectively, indicating that the water column was not thermally stratified during the study period. At 5 m depth, the temperature ranged from a minimum of 20.0 °C before sunrise to 20.9 °C at 15:00. Maximum and minimum seawater temperature at 20 m depth occurred at the same hours, with values between 19.7 and 20.5 °C.

### Photosynthesis and photoprotection

The photochemical efficiency of PSII (ΔF/Fm’) showed a similar daily pattern at the two depths, with similar values at every sampling time (Fig. 1B; Supplementary Table S1). Maximum ΔF/Fm’ values were found before sunrise and progressively decreased until midday, when the lowest values were reached. Later in the day, as irradiance started to decrease, photochemical efficiency progressively increased to equally maximum levels around dusk. The ΔF/Fm’ reduction from sunrise to high-light hours (12:00 to 15:00) was about 80% both at 5 and 20 m depth. The quantum yield of regulated non-photochemical quenching (NPQ) showed an opposite pattern to that observed for ΔF/Fm’, with no significant differences between the two depths (Fig. 1B). The lowest values of NPQ were measured before the sunrise and the highest around midday, with similar values at 5 m (0.403 ± 0.05) and 20 m (0.471 ± 0.01), which were twice the minimum ones.

As a consequence of the similar ΔF/Fm’ pattern between plants at the two depths and the higher light availability in the shallow meadow portion, electron transport rates (ETRs) of shallow plants during the light hours were significantly higher than deep plants (Fig. 1C). Their daily integrated photosynthetic electron transport rate was on average 5-fold higher than that at 20 m depth. The ETRs of deeper plants held their maximum values for longer (from 09:00 to 15:00) than shallow plants (from 09:00 to 12:00) (Fig. 1C). This pattern of delayed response of deeper plants was also observed in the expression of genes related to photosynthesis and photoprotection (see below).

No significant differences were found between depths or among times of the day in the leaf concentrations of both chlorophylls and carotenoids (Table 1). The ratio between chlorophyll *a* and *b* was around 2. Violaxanthin (V) and zeaxanthin (Z) concentrations were one order of magnitude higher than anteraxanthin (A). On the other hand, the de-epoxidation index was significantly higher at −5 m than at −20 m but there were no significant differences between morning and midday.

### Respiration

**Figure 2:**
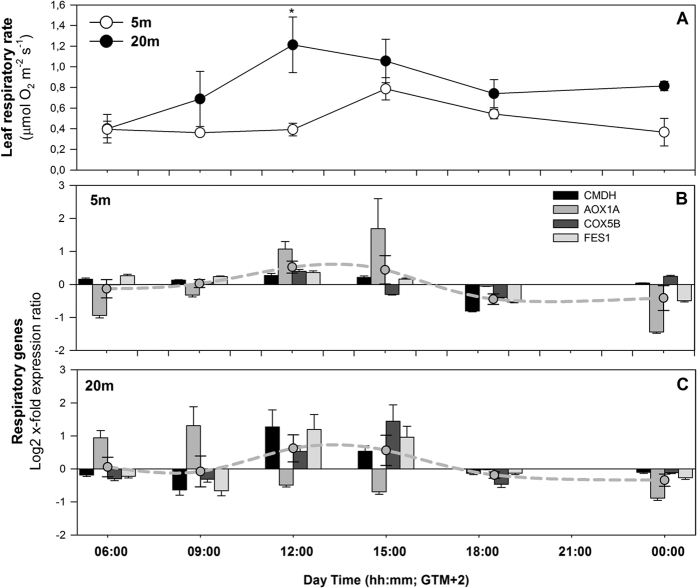
Daily cycle of leaf respiration (**A**) and of mRNA expression of respiration-related genes of shallow (**B**) and deep (**C**) *P. oceanica* plants. Relative expression levels at given time points are calculated over their daily average expression. Dashed lines represent the average expression level of all analyzed genes. Asterisks indicate significant differences among shallow and deep plants at a given sampling time. *p < 0.05.

The respiratory activity of *P. oceanica* leaves was not significantly different between the shallow and deep plants, except at 12:00, where it was higher in the deep site (Fig. 2A). Leaf respiration showed similar diel rhythms at the two depths, with an overall increase at high-light hours and reduced rates at lower irradiances and during the night (Fig. 2A). The response of leaf respiration to light of shallow plants was delayed in respect to deeper plants. The leaf respiration of deeper plants peaked from 09:00 to 12:00 whereas in shallow plants the peak was from 12:00 to 15:00 (Fig. 2A). The lowest leaf respiration for both −5 and −20 m plants occurred before sunrise, at 06:00. The shallow plants showed lower respiratory rates than the deep plants during morning samplings (09:00 and 12:00), with significant differences only at 12:00 (Fig. 2A). Leaf respiration was positively correlated with PAR irradiance levels (n = 6, r = 0.85, p = 0.034; Supplementary Table S2) only in deep plants.

### Gene expression

#### Daily mRNA variation in shallow and deep plants

**Figure 3:**
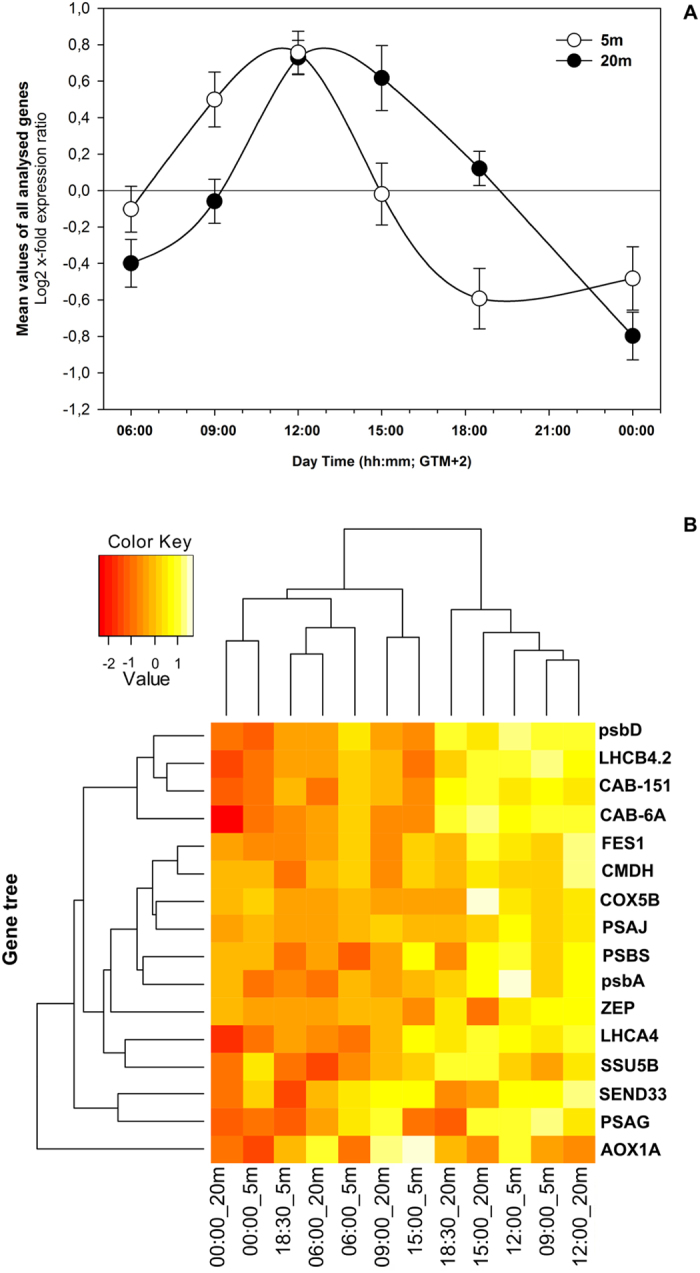
Average daily expression patterns of all genes of interest in shallow (white circles) and deep (black circles) *P. oceanica* plants (**A**); and Heatmap (**B**) showing cross-correlation among experimental conditions and similarity of gene expression profiles of the sixteen selected genes analyzed by RT-qPCR. X-axis: columns display the cDNA from the six collection times at −5 and −20 m depth, clustered by similarities; y-axis: each row displays the expression level of a given gene in the corresponding cDNA, clustered by similarities across experimental conditions. Color key (white: highest expression strength; red: lowest expression strength).

*P. oceanica* plants exhibited a dynamic daily regulation of mRNA levels of selected genes of interest (GOIs). The averaged expression pattern for all analyzed genes indicated a similar daily trend in plants from the shallow and deep meadow portions, but with a general delay in the deepest stand (Fig. 3A). In shallow plants, GOIs mRNA accumulation started soon after the light onset, peaked around noon (PAR = ~300 μmol m^−2^ s^−1^), and then progressively decreased until dusk, with low values during the night (Fig. 3A). In plants from −20 m, transcriptional activation of GOIs was delayed by about 3 hours. Activation started at 09:00 and attained maximum expression values at 12:00 and 15:00 under highest PAR values. At 18:30 GOIs were still up-regulated with respect to their daily average expression, in contrast to that observed in shallower plants that were down-regulated at this time (Fig. 3A).

Hierarchical clustering (Fig. 3B) identified two main clusters corresponding to higher and lower expression levels, and further highlighted the time shift between shallow and deep plants. For example, the −20 m plants at 09:00 (09:00_20 m) belong to the low expression group, while 09:00_5 m falls within the high-expression group and in particular branches with 12:00_20 m. Similarly, plants of 18:30_20 m still cluster with the high-expression group, while 18:30_5 m is included in the low-expression group and clusters with 06:00_20 m.

Most of the photosynthesis-related genes analyzed showed a synchronized diel pattern of expression at each depth (Fig. 1D,E). The expression of some genes was delayed in relation to others, such as *LHCA4* and *SSU5B* at 06:00 and *SSU5B, LHCA4* and *SEND33* at 15:00, at −5 m. *PSAG* at −20 m was faster to react at 09:00 and at 18:30 (together with *SEND33*) than the other photosynthetic genes (Fig. 1D). *PSAJ* and *psbA*, encoding for subunits of PSI and PSII, respectively, and *LHCA4* showed significant correlations with chlorophyll *a* fluorescence parameters. Negative correlations were observed with the photochemical efficiency of plants (ΔF/Fm’; n = 12, p < 0.05) and positive with the non-photochemical quenching (NPQ; n = 12; p < 0.05) and ETRs (n = 12, p < 0.001 and n = 12, p < 0.001, respectively).

A delayed response of photoprotective genes *PSBS* and *ZEP* (Fig. 1D,E) in deeper plants in relation to shallow plants was also observed, as in the case of the photosynthetic genes referred above. *PSBS* expression was positively correlated with PAR (n = 12, r = 0.77, p = 0.003; Supplementary Table S2) and with the dissipative process of non-photochemical quenching NPQ (n = 12, r = 0.85, p < 0.001), at both depths.

The average expression of respiration-related genes showed a similar diel cycle at the two depths, increasing with light to maximum values at 12:00 to 15:00 hours (Fig. 2B,C) and decreasing to lowest levels during the night. *AOX1A* showed the highest diel variation, especially at 5 m depth. At −20 m, its expression trend deviated from that of the other respiratory genes, being strongly up-regulated in the early morning (06:00 and 09:00), and down-regulated in the following hours, when *CMDH, COX5B* and *FES1*started to be expressed. In shallow plants, the expression peak of this gene occurred later (12:00–15:00) in line with the subsequent rise in respiration (Fig. 2A–C). The level of expression of the *AOX1A* gene of shallow plants was significantly correlated with light (n = 6, r = 0.89, p = 0.018; Supplementary Table S2) in contrast with the other genes. In deep plants, the genes *CMDH* and *FES1* were positively correlated with light (n = 6, r = 0.835, p = 0.042 and n = 6, r = 0.83, p = 0.041, respectively) similarly to the leaf respiratory rates, as described above.

**Figure 4:**
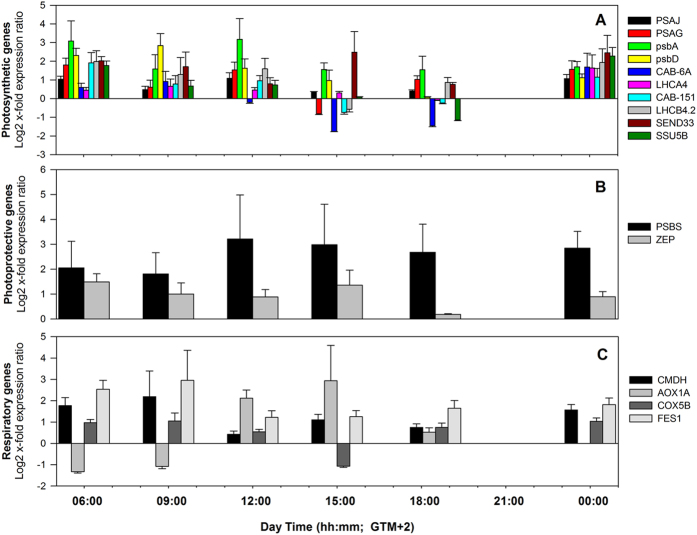
Relative quantification of photosynthesis (**A**), photoprotection (**B**) and respiratory-related (**C**) genes, in shallow *P. oceanica* plants (−5 m) at given time points, considering gene expression in deep plants (−20 m) as control condition. Relative quantification was obtained using REST 2009^96^. Significant results with corresponding *P(H1)* values are reported in Table S3.

Looking at differences in expression of GOIs within each sampling time, plants from −5 m showed a stronger induction in respect to −20 m plants (Fig. 4; Supplementary Table S3). Only after midday and at dusk (15:00 and 18:30) some genes were more expressed in the deep than in the shallow meadow (e.g. *CAB-6A* and *SSU5B*). Transcripts for PSII subunits such as *psbA, psbD* and *PSBS* were among those displaying the strongest up-regulation in shallow plants, especially during light hours (Fig. 4; Supplementary Table S3). Chlorophyll *a/b* binding proteins were also generally more expressed in shallow plants, although significant results were only found for those associated with PSII (LHC II) (Fig. 4; Supplementary Table S3). The ferredoxin-1 encoding gene *SEND33* showed large differences in expression levels between plants from the two depths (Fig. 4; Supplementary Table S3). Respiration-related genes, *CMDH, COX5B* and *FES1*, were over-expressed in −5 m plants at almost all points of time (Fig. 4; Supplementary Table S3). *AOX1A* exhibited a different trend, being down-regulated at −5 m in the early morning (06:00 and 09:00), although not significantly (Fig. 4; Supplementary Table S3).

#### Daily protein variation in shallow and deep plants

Almost 80 protein bands were detected in each gel by image analysis, 15 of which showed significant variation during the light hours (Supplementary Table S4). Accession, predicted protein sequences and NCBI Blastp against non-redundant protein sequences are reported in Supplementary Table S5. Most of these were structural proteins and enzymes involved in key steps of the light-dependent reactions of photosynthesis (e.g. PSI and PSII subunits, chlorophyll *a/b* binding proteins, chloroplastic ATP synthase), carbon fixation (RuBisCO and RuBisCO activase), and glycolysis (phosphoglycerate kinase, glyceraldehyde 3-phosphate dehydrogenase).

**Figure 5:**
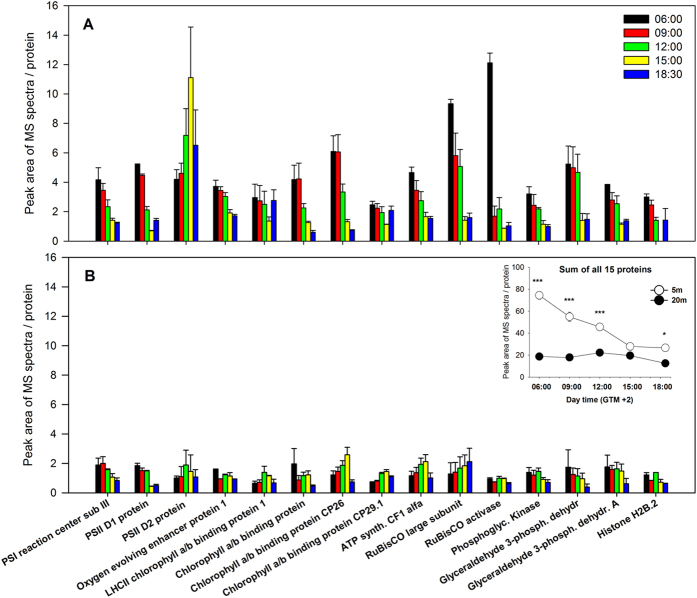
Daily expression pattern of accumulation of 15 proteins of *P. oceanica* at 5 m (**A**) and 20 m (**B**) depth. Inner panel represents the daily pattern of the sum of all proteins. Asterisks indicate significant differences among shallow and deep plants at a given sampling time. *p < 0.05; **p < 0.01; ***p < 0.001.

The overall daily pattern of identified proteins revealed strong differences among plants from the two depths (significant D × T interaction for 9 out of the 15 proteins analyzed, Supplementary Table S6). Shallow plants exhibited a significant and progressive reduction in protein abundance, with maximum accumulation before sunrise (06:00) and in early morning (09:00) and lower values during the afternoon and just after sunset (18:30) (Fig. 5). RuBisCO large subunit and RuBisCO activase displayed the highest concentrations at 06:00, but their levels underwent a huge decline just after sunrise. A group of proteins formed by glyceraldehyde 3-phosphate dehydrogenase proteins, Phophoglycerate kinase and oxygen-evolving enhancer protein 1 were very abundant until noon, significantly decreasing thereafter until the sunset. A different trend was observed for the photosystem II D2 protein, peaking at 15:00 with 5-fold significantly higher concentrations than morning samples (06:00 and 09:00). Chlorophyll *a/b* binding protein of LHCII type 1 and the chlorophyll *a/b* binding protein CP29.1 conversely showed a significant reduction at 15:00, and similar concentrations for the rest of sampling times (Fig. 5).

Deep plants presented lower leaf concentrations of all analyzed proteins than shallow plants and the patterns of variation throughout the day were not as consistent as in shallow plants (Fig. 5). Protein concentration was similar all along the day except for PSII D1 that exhibited significantly lower concentrations at 15:00 and 18:30 with respect to previous hours.

The PSII D2 protein was the only one showing a significant and positive correlation with PAR values (n = 10, r = 0.73, p = 0.015; Supplementary Table S2).

#### Genetic diversity and structure

**Figure 6:**
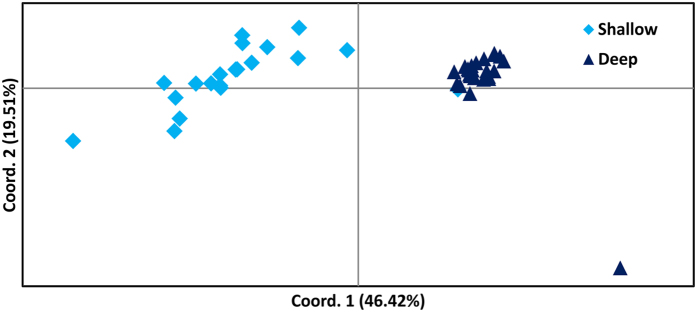
PCoA analysis based on the genetic distance matrix, for the two depths (−5 m and −20 m) of the STARESO *P. oceanica* meadow. The light and dark blue symbols correspond to genotyped individuals from shallow and deep site, respectively. Percentages of variation explained by the first two axes are 46.42 and 19.51, respectively.

Pair-wise comparison of F_ST_ estimates between the shallow and deep sites (F_ST_ = 0.169) indicated high genetic differentiation related to depth. The shallow site showed higher genotypic richness, percentage of polymorphic loci, total number of alleles and average number of alleles per locus, compared to the deep site (Supplementary Table S7). Seventeen private alleles were present at the shallow site, while only two were present at the deep site (Supplementary Table S7). Observed heterozygosity (H_o_) was always higher than expected (Supplementary Table S7), with several loci (15 in shallow plants, 14 in deep plants) deviating significantly from the Hardy-Weinberg equilibrium (indicated with asterisk in Supplementary Table S8). Analyses of Molecular Variance (AMOVA) was performed partitioning the molecular variance into two levels (among depths and within depths), and almost 50% of the total variation was explained by the between-depths component (48%; Supplementary Table S9). Principal Coordinate Analysis (PCoA), with the first axis explaining 46.42% of the total variance, clearly separates the meadow into two clusters corresponding to the two depths (Fig. 6).

## Discussion

Our results revealed for the first time not only that diel oscillation of *P. oceanica* photophysiological and respiratory responses were related to transcriptional responses and protein expression of the genes involved in those processes, but also that there was a response asynchrony between shallow and deep plants, probably due to strong differences in the light environment.

The daily patterns of fluorescence-derived parameters indicate a dynamic regulation of the photosynthetic process in *P. oceanica*^19^,^20^. As light increases during the day, plants activate photoprotective mechanisms, decreasing photochemical efficiency. This photosynthetic downregulation was similar across depths, and recovered fully by nightfall, suggesting that plants did not experience accumulated photodamage at the light conditions experienced in this time of the year. This finding contrasts with results obtained in the seagrass *Halophila stipulacea* at similar light levels, where photochemical efficiency of shallow plants at 8 m depth did not recover by the end of the day, but showed a gradual increase overnight indicating the longer term activation of recovery processes^21^.

The NPQ induction, the decrease in photochemical efficiency and the upregulation of the photoprotective genes observed both in shallow and deep plants of *P. oceanica* during high-light hours suggest the triggering of energy dissipation mechanisms such as the xanthophyll cycle^29^. The de-epoxidation index of *P. oceanica* was significantly higher in shallow than in deep plants, indicating higher energy dissipation through the xanthophyll cycle. This corresponds to the up-regulation of photoprotective genes in shallow plants, as compared to deep ones, and reveals that *P. oceanica* has the potential needed for photoprotection in the highly transparent waters of Stareso. However, we did not detect differences in the de-epoxidation index between dawn and midday both in shallow and deep plants, due to high retention of zeaxanthin before sunrise. A possible explanation is the onset of a process called sustained dissipation, which refers to thermal dissipation that is not dependent on the trans-thylakoid ΔpH as for the xanthophyll cycle, and does not relax rapidly in darkness^30^,^31^. It involves the continuous engagement of zeaxanthin and its dark retention and is the reason of decreased photosynthesis caused by high stress^30^,^31^,^32^. Despite this, NPQ increased during the day due to other quenching mechanisms such as those involving PsbS. The accumulated zeaxanthin may actually prompt NPQ induction allowing a faster response to high-light conditions^32^,^33^.

Interestingly, the mRNA of the photosystem II subunit S (*PSBS*) exhibited a daily oscillation highly and positively correlated with the process of non-photochemical quenching at both depths. This points towards the important role of PsbS in activating NPQ mechanisms in seagrasses, as described for terrestrial plants^34^,^35^,^36^,^37^. Photosynthesis is one of the most fundamental processes regulated by circadian clocks at both the metabolic and gene-expression levels^38^,^39^,^40^. Accordingly, mRNAs of most photosynthesis-related components, including *cab* genes encoding chlorophyll *a/b* binding proteins, and subunits of photosystems I and II, followed the daily pattern of photophysiological parameters and exhibited a clear diurnal rhythmicity in plants from both depths, with increasing levels slightly before or after sunrise, peak abundance around noon, and decreasing expression at dusk and night. In higher plants, the right timing and amplitude of expression patterns of temporally regulated genes are determined by a combination of endogenous rhythms and external signals, primarily light and temperature, that control transcription^41^,^42^,^43^. Our data do not allow establishing the existence of endogenous rhythms, but the observed positive correlations with daily irradiance levels for some functional and structural photosynthetic genes indicate the important role of external signals.

In the chloroplasts of terrestrial plants and green algae, the expression of the *psbA* gene is regulated via a complex network including transcriptional control, regulation of mRNA stability, and the control at the level of translation^44^. The interdependence between irradiance and the expression of *psbA* that encodes the PSII D1 core protein observed here, reflects the functioning of the multistep PSII repair cycle process^45^,^46^,^47^,^48^. Increasing damage of the PSII D1 protein with enhanced irradiance forces plants to continuously repair the impaired PSII to maintain an efficient photosynthetic process^44^,^49^. Our analysis revealed a different timing for PSII D1 protein and *psbA* mRNA accumulation during the day. In shallow plants, maximal abundance of PSII D1 protein was detected in the early morning, and then a progressive decline occured, reflecting its consumption in repairing PSII along the day. Differently, the peak of *psbA* mRNA accumulation was detected at midday, possibly to sustain the processes of light-induced degradation and *de novo* biosynthesis of the relative protein in high light, which occurr simultaneously during PSII repair.

Due to the highly oxidative chemistry of water splitting mediated by the oxygen-evolving complex, the PSII extrinsic protein OEE1 (Oxygen-evolving enhancer protein 1) can be released from its binding site, thereby disorganizing PSII and impairing the complex^50^,^51^. The OEE1 subunit of PSII was also found up-regulated in the shallow *P. oceanica* plants, likely promoting the protection and recovery of the PSII complex and enhancing the rate of electron transport under high-light conditions. In fact, the expression of the nuclear gene encoding OEE1 is required for high levels of photosynthetic oxygen evolution^52^ and OEE1 may be involved in the regulation of the PSII^53^.

In general, the higher transcription rate found in shallow plants of *P. oceanica* is reflected in the higher accumulation of proteins. This is supported, for example, by the significant and strong up-regulation of *cab* and photosystems-associated transcripts, which corresponds to a higher translation and turnover of the related proteins. Particularly, the differential rate in translation of proteins of the PSII complex seems to drive *P. oceanica* acclimation to the light environment and to be the main metabolic pathway for depth acclimation^12^,^13^,^14^. Higher translation and protein synthesis rates of shallow plants are also evident by the peaks of RuBisCO and RuBisCO activase proteins just before sunrise. In the presence of light and at an optimal temperature, RuBisCO activase promotes the release of the inhibitor ribulose 1,5-bisphosphate from the catalytic sites of RuBisCO, thus activating the enzyme. This daily pattern is in agreement with the circadian oscillations of RuBisCO activase found in tomato^54^, apple^55^, and *Arabidopsis*^56^. The maximal abundance of RuBisCO protein in shallow plants was shifted forward about seven hours in respect to the corresponding transcripts (*SSU5B*), suggesting that translation of RuBisCO subunits occurs during the night so that by sunrise the plants are ready to take maximum advantage of available light for photosynthesis. During the day, RuBisCO level drastically decreased to a minimum at sunset. In the deep site there was a strong down-regulation of *P. oceanica SSU5B* transcripts during the night that explains the low level of RuBisCO protein at sunrise. The up-regulation of *SSU5B* during light hours is coherent with the increasing RuBisCO level along the day.

On what concerns the cellular energetic metabolism, it was observed that the level of Glyceraldehyde 3-phosphate dehydrogenase (GADPH), the enzyme that catalyzes the first step of the third stage of glycolysis, was highest at sunrise in both shallow and deep plants, to maintain the energy balance within the cells before photosynthesis was triggered. The enzyme Phosphoglycerate kinase, which also participates in the maintenance of the organic and inorganic carbon balance, showed the same pattern of variation as GADPH. This enzyme catalyzes the transfer of a phosphate group from 1,3-bisphosphoglycerate in the first ATP-generating step of the glycolytic pathway. In the opposite direction, it also generates ADP and 1,1,3-bisphosphoglycerate in gluconeogenesis. These processes modulate the respiratory responses, both at the gene expression and at oxygen consumption levels, which were highest from 12:00 to 15:00 both in shallow and deep plants.

The seagrass *Zostera marina* also revealed varying respiration rates on a diel basis, even though the peaking times of respiration differed from that of *P. oceanica* (Rasmusson *pers. comm.*). The substantial change of the respiratory activity over the day in both seagrass species supports the hypothesis that respiration should not be considered constant when assessing and modeling carbon and oxygen fluxes for vegetated marine habitats. A similar respiration rate between plants at different depths has also been shown for other seagrass species. In *Thalassia hemprichii* and in *Zostera marina* respiration was not significantly different at two different depths, even though the photosynthetic rate was higher in the shallow plants^57^,^58^. In our study, although the diel trend in respiration was comparable among plants from the two depths, a phase shift was present, with shallow plants increasing their leaf respiratory activity later than deep plants. While deep *P. oceanica* plants presented a peak in respiration around noon, shallow plants had their highest respiratory activity from 15:00 to 18:30. At the two depths, the high photosynthetic production of carbohydrates in high-light hours, could induce an elevated level of respiration, since both processes are tightly interdependent^59^. At those hours, up-regulation of malate dehydrogenase (*CMDH*), an important enzyme of the tricarboxylic acid cycle^60^ and *COX5B* and *FES1*, regulating the cytochrome electron transport^61^,^62^, was observed, indicating that the respiratory machinery was working to its full extent. In the shallow plants the up-regulation of *AOX1* suggests an over-excitation of the mitochondrial electron transport chain since this gene controls the alternative oxidase that is working as a safety mechanism redirecting the flow of excess electrons^63^,^64^.

The present study revealed coherent daily oscillations in *P. oceanica* at different organizational levels (i.e. gene transcription, protein expression and photosynthesis and respiration responses) whose peaks occurred at different timings in shallow and deep plants. The acclimation of key biological processes to different light availability cycles ensures the optimal exploitation of light resources that explains the wide bathymetric distribution of this species. Small depth differences in photoperiod and larger differences in irradiance levels and light spectra are likely to have a profound influence on observed oscillations of gene expression and must be responsible for the asynchrony observed between depths. Changes in light quality and quantity affect the central clock’s synchronization and periodicity of functions^65^. Different organisms respond differently to changes in irradiance, shortening or lengthening the periodicity^66^,^67^,^68^. Yet, light spectral composition, which implies cardinal roles for the different classes of photoreceptors, can synchronize diverse metabolisms over the diel phases, influencing the time period and the clock phase^66^,^69^,^70^. The whole issue of circadian regulation of biological function is still unexplored in *P. oceanica*, and requires ad hoc experimental conditions.

Our results also showed that the shallow and deep *P. oceanica* portions of the studied meadow are genetically differentiated, with a higher genetic and genotypic diversity at the shallow site. Genetic differentiation between stands growing at different depth is known in *P. oceanica*^5^,^71^, and it remains to be demonstrated whether this is related to the pressure of contrasting environmental cues that require species acclimation strategies as revealed here. A recent study reported different acclimation strategies and possibly local adaptation in plants at different depth, as response to different temperature^72^.

Although limited to a single autumn day, this is to date the most complete description of the daily physiological performance of a seagrass species under natural conditions, and offers for the first time converging multilevel evidence of the existence of diurnal oscillations in key processes governing the functioning of a seagrass meadow along its bathymetric distribution. The photochemistry pathway of energy use was more effective in shallow plants due to higher light availability, but those plants needed more investment in photoprotection and photorepair, requiring higher translation and protein synthesis than deep plants. The genetic differentiation between deep and shallow stands suggests the existence of locally adapted genotypes to contrasting light conditions. Our findings also emphasise the importance of considering daily fluctuations in photosynthetic and respiratory processes (from the molecular to the physiological levels) and the intrinsic differences between shallow and deep plants, in the management and conservation of these key coastal ecosystems.

## Methods

### Study site and sampling design

The study site is located in the Revellata Bay, at the west side of the Calvì Bay (Corsica, Mediterranean Sea), where a dense *P. oceanica* meadow extends from 3 to 37 m depth, in front of the STAtion de REcherches Sous-marines et Océanographiques (STARESO) (8°45E, 42°35N). The area is considered as a Low Nutrient-High Chlorophyll system^73^, with exceptionally low anthropogenic disturbances. The Calvì Bay serves the status of reference body of water for the northwestern Mediterranean, according to the PREI (*Posidonia oceanica* Rapid Easy Index) method applied on existing *P. oceanica* meadows^74^,^75^,^76^. *P. oceanica* samples were collected by SCUBA diving six times over a 24 hour period at 06:00, 09:00, 12:00, 15:00, 18:30 and 00:00 (GMT+2), at 5 and 20 m depth, in mid-October 2011.

### Irradiance and *in situ* chlorophyll a fluorescence

Measurements of variable fluorescence were obtained with submersible modulated fluorometers (Shutter Fluorometer, Aquation Pty Ltd, Australia), deployed within the meadow at 5 and 20 m depth, minimizing alterations in the canopy structure. *P. oceanica* leaves were positioned in the sample holders of the fluorometers so that a portion of leaf halfway along the blade was examined. Accounting for the typical pattern of variation among and within seagrass leaves, as function of tissue age^23^,^77^,^78^, measurements were taken on the same leaf portion (15–25 cm above the leaf ligule) on the first mature leaf of randomly selected shoots. Prior to measurements, epiphytic material was gently removed.

The fluorometers were programmed to remain open between measurements to expose the leaf to ambient light, then at each time point the shutter closed over the sample leaf and exposed it to a saturating light pulse followed by a 10 s exposure to far red light (735 nm; to measure Fo’), before applying another saturating pulse measurement. Following these measurements, the shutter then opened and remained so until the next time point. From these variable fluorescence measurements we calculated the maximum and effective quantum yields of PSII photochemistry (ΔF/Fm’), and the quantum yield of photosynthetic downregulation (NPQ)^21^,^79^. Electron transport rates (ETR) were calculated according to ref. 80 using ambient PAR values measured at the same location and time. Absorptance values were estimated from leaves collected at the site according to ref. 81, and we assumed equal sharing of photons between photosystems I and II for ETR calculation. See ref. 28 for more details of these measurements. Photosynthetically active radiation (PAR) was measured using the PAR sensor of each Shutter Fluorometer. At each sampling depth and time, four independent measures were taken (n = 4).

### Photosynthetic pigments

Shoot samples for the analysis of pigments were collected only before sunrise (06:00) and at midday (12:00), and brought in darkened containers to the surface. Leaves were cleaned of epiphytes, rinsed with distilled water and rapidly frozen at −80 °C. Time from sampling to storage was kept at the minimum (about 5 min). A quantity of 100 mg frozen leaf material was ground in liquid nitrogen in the presence of sodium ascorbate. Pigments were extracted in 5 mL 100% acetone buffered with CaCO3^82^. The extracts were sequentially filtered with LS 5.0 mm membrane filters and hydrophobic PTFE 0.2 mm filters and stored in the dark at −20 °C prior to analysis. The extraction took place under low-light conditions. Chlorophyll *a* (Chl*a*) and *b* (Chl*b*) were quantified by spectrophotometric absorbance reading, using the equations of ref. 83. Carotenoids were separated and quantified in an isocratic High Performance Liquid Chromatography (HPLC), as described in ref. 84 after^85^. Chlorophylls were expressed per square meter of leaf area and all carotenoids were expressed on a total chlorophyll basis. The de-epoxidation index was calculated as (A + Z)/(V + A + Z), where V is violaxanthin, A is anteraxanthin, and Z is zeaxanthin^29^.

### Leaf respiratory rates

*P. oceanica* shoots from selected depths and times were immediately brought in darkened containers to the laboratory for respiratory measurements. Epiphyte-free leaf segments of ca. 2 cm^2^ were incubated in darkness in three 3 ml chambers connected to Clark-type oxygen electrodes (DW1/AD, Hansatech, England). Leaves were allowed to acclimate for 15 minutes until steady state respiration was reached. The temperature in the chambers was regulated by water from a temperature-controlled tank, flowing through jackets surrounding the chambers, keeping the temperature at 21 ± 1 °C. Natural seawater from the collection site with a pH ranging from 8.08 to 8.25 and a salinity of 38^86^ was used and renewed for each measurement.

### mRNA and protein expression

For mRNA and protein expression analysis, entire shoots were collected and brought in dark containers from the sampling site to the laboratory (within 5 min), where they were rapidly scraped free of epiphytes, towel-dried and either stored in RNAlater© tissue collection (Ambion, Life Technologies) (samples for RNA extraction, n = 3) or frozen in liquid nitrogen (samples for protein extraction, n = 5). Only the youngest fully mature leaves of the shoot were selected.

#### RNA extraction and RT-qPCR

The RNA extraction procedure is detailed in ref. 28. RNA quantity and purity were assessed by Nanodrop (ND-1000 UV-Vis spectrophotometer; NanoDrop Technologies), and RNA quality was checked by 1% agarose gel electrophoresis. Total RNA (500 ng) was retro-transcribed in cDNA with the iScript™ cDNA synthesis kit (Bio-Rad). Sixteen Genes of Interest (GOIs) were selected according to their role in photosynthesis, photoprotection and respiration (cf. ref. 28; Table 2), and their expression profiles were evaluated by RT-qPCR. For a detailed description of primer design and PCR optimization procedures see refs 87,14. Primer’s sequences, efficiencies (*E*) and correlation coefficients (R^2^) can be retrieved from Table 2. PCR efficiencies for all primer pairs were always >92% and all R^2^ were >0.96. RT-qPCR reactions were conducted in triplicate to capture intra-assay variability and each assay included three no-template negative controls for each primer’s pair^28^. A 1:100 cDNA template dilution was used, in order to allow almost all gene amplifications to fit in the optimal detection window (from 15 to 25 cycles). The gene encoding for the ribosomal protein L23 (GenBank: GO347779) served as reference gene in our assays, which has been previously tested in different light conditions^87^. Gene expression data are shown as log2 expression ratio ± SE. To visualize gene expression patterns, a heatmap was generated in R using the heatmap.2 function from the gplots package (http://CRAN.R-project.org/package=gplots).

#### Protein extraction and 1-DE electrophoresis

Total proteins were extracted following the protocol in Spadafora *et al*.^88^, which is specifically adapted to marine plants, at all time points except 00:00. As the leaf tissues of marine plants are generally rich in secondary metabolites, such as phenols, disaccharides, and lipids, which interfere with protein extraction and purification^88^,^89^, the protocol involves the use of trichloroacetic acid. This strong acid precipitates proteins from the tissue powder and washes out phenols, sugars and other soluble molecules when dissolved in the acetone. For each extraction, 1.4 g of leaves were utilized. Proteins were quantified by the Bradford assay. Protein yield was measured as mg of protein per g fresh tissue weight.

Gel preparation, run and staining were performed as in ref. 90. Each lane of the same SDS-PAGE was divided into six slices from 200 to 10 kDa and manually excised from the gel, cut in small pieces, S-alkylated and digested overnight at 37 °C with trypsin^91^. Digested peptides were extracted from the gel slices and processed using mass spectrometry (LC-MS/MS) with the LTQ-Orbitrap XL (Thermo Fischer Scientific) as fully described in ref. 90. Spectra acquired by LC-MS/MS were used to identify peptide sequences using PEAKS™ Studio 6 software (http://www.bioinfor.com/peaks-inchorus, Bioinformatics Solutions, Inc.) against a customized database built with a collection of protein sequences from multiple databases as described in ref. 92, and from the transcriptome sequences of the NGS Illumina dataset (unpublished data). The differential expression of proteins at different depths and times were evaluated by the labeling-free approach using the peptide ion peak area measurements^93^. Multiple gel slices from each sample were pairwise combined at corresponding ranges of molecular weight to detect differentially expressed proteins at each time and depth. The peptide ion peak area measurements of MS/MS spectra were quantified by PEAKS™ Q tool for label free quantification. The family of proteins can be identified as long as one protein is present in the sample; families were clustered together to avoid false positives.

### Statistical analyses

Results are presented as mean values ± standard error of replicate samples, unless indicated otherwise. One-way repeated-measures ANOVA was used to assess the statistical significance of depth (as between- subject factor) on the diel trends of chlorophyll *a* fluorescence parameters (as within-subject factor). Rm ANOVAs were carried out according to the procedures described in ref. 94. Before running the analyses, the variance-covariance matrices were tested for sphericity using Mauchly’s test, and if the assumption was not met (p < 0.05) the Greenhouse-Geisser (G-G) epsilon adjustment was applied to the degrees of freedom, as it is considered a conservative correction to reduce Type I error^94^. Student-Newman-Keuls’ post-hoc analysis were done to identify significant differences (p < 0.05) among factor levels.

Two-Way ANOVAs were used to test main and interactive effects of depth and time at a significance level of p < 0.05, on pigments, leaf respiration and protein expression. The Student-Newman-Keuls post-hoc test was applied to reveal significant differences between individual means^95^. Statistical analyses were performed using the statistical package STATISTICA (StatSoft Inc, v. 7.0). For mRNA expression data within each sampling time, significant differences in mRNA levels between shallow and deep plants were assessed using the hypothesis test *P*(*H1*) implemented in REST 2009^96^. This software provides proper error propagation and robust statistical analysis by using a random reallocation algorithm with 10,000 iterations. The direct effect of irradiance on *P. oceanica* responses measured at different plant levels (i.e. mRNAs, proteins, respiration and photochemistry), was estimated through Pearson correlation analyses. Correlations among the different parameters were also performed.

### Genetic diversity

A total of 51 *P. oceanica* shoots were collected at the two depths, at a reciprocal distance of 5–6 m to minimize the risk of sampling within the same clonal patch. Leaf material (about 5 cm) from individual samples was cleaned from epiphytes and dried in silica gel prior to DNA isolation. Genomic DNA was extracted using the NucleoSpin® 96 Plant II kit (Macherey-Nagel) following the manufacturer’s instructions. All samples were genotyped at 29 microsatellite loci, 13 putatively neutral^97^,^98^ and 16 EST-linked^99^. Microsatellites were amplified in multiplex PCR reactions and scoring was performed following^5^,^99^.

All genetic diversity indices were calculated both at population and depth levels. Genotypic richness was estimated as *R* = G − 1/N − 1 where G is the number of distinct genotypes and N is the number of individuals. Calculations were performed using the GenClone software^100^. All subsequent analyses were performed eliminating all but one individual from replicated genotypes. Heterozygosities and F_IS_ values were calculated eliminating the chloroplastic locus (Poc-*trn*). The software GenAlEx^101^ was used to estimate standard population genetic statistics such as the number of alleles (A), number of alleles per locus (A/locus), private alleles (PA), observed (H_o_) and unbiased expected heterozygosity (uH_e_). The exact test of Hardy-Weinberg equilibrium for each locus was performed using the software Arlequin ver. 3.5^102^. F_IS_ and F_ST_ values were calculated using the software FSTAT^103^. P values for pair-wise tests of differentiation were obtained after 1000 permutations. The nominal level for multiple tests was set to 0.001. To assess the component of genetic variation due to habitat contrasts, an Analysis of Molecular Variance (AMOVA) was carried using GenAlEx ver.6.5^101^. To visualize how the genetic characteristics of each individual were organized in a multidimensional space based on allelic data, Principal Coordinates Analysis (PCoA) was performed using the software GenAlEx^101^.

## Additional Information

**How to cite this article**: Procaccini, G. *et al*. Depth-specific fluctuations of gene expression and protein abundance modulate the photophysiology in the seagrass *Posidonia oceanica* *Sci. Rep.* **7**, 42890; doi: 10.1038/srep42890 (2017).

**Publisher's note:** Springer Nature remains neutral with regard to jurisdictional claims in published maps and institutional affiliations.

## Supplementary Material

Supplementary Material

## Figures and Tables

**Table 1:** *Posidonia oceanica* pigment content at 5 m and 20 m depth at 06:00 and 12:00. Values represent means ± standard error.

	−5 m		−20 m	
	06:00 (n = 5)	12:00 (n = 6)	06:00 (n = 9)	12:00 (n = 9)
Chlorophyll *a* (Chl*a*)	30.3 ± 8.9	46.5 ± 9.5	54.6 ± 5.2	53.3 ± 8.5
Chlorophyll *b* (Chl*b*)	16.6 ± 5.6	22.8 ± 4.7	28.6 ± 2.9	26.0 ± 4.3
Total Chlorophyll (ChlT)	46.9 ± 14.4	69.3 ± 14.2	83.2 ± 7.9	79.3 ± 12.8
Chl*a*/Chl*b*	1.963 ± 0.146	2.087 ± 0.085	1.917 ± 0.054	2.053 ± 0.053
Violaxanthin (V)	35.2 ± 9.9	43.8 ± 3.0	43.3 ± 4.6	45.5 ± 3.0
Anteraxanthin (A)	2.226 ± 0.808	3.963 ± 1.111	2.944 ± 0.866	3.129 ± 0.390
Zeaxanthin (Z)	80.5 ± 28.9	86.7 ± 8.9	34.4 ± 8.7	76.5 ± 21.8
(V + A + Z)/ChlT	117.9 ± 39.1	134.4 ± 6.9	80.6 ± 8.2	125.1 ± 23.7
(A + Z)/(V + A + Z)*	0.686 ± 0.043	0.669 ± 0.076	0.427 ± 0.067	0.546 ± 0.073

*Indicates significant differences between depths. Chlorophylls are expressed per square meter of leaf area (μmol m^−2^) and all carotenoids are expressed on a total chlorophyll basis (mmol mol^−1^ ChlT).

**Table 2:** Genes of interest and reference gene used in *Posidonia oceanica* RT-qPCR assays. Gene name and symbol, GenBank Accession Number, biological process, product size (S, base pair), percent efficiency (*E*), correlation coefficient (R^2^) and primer sequences are shown.

Gene name	Symbol	Acc. no.	Biological Process	S	*E*	R^2^	Primer sequence 5′- 3′
60 s ribosomal protein L23	L23	GO347779	Translation	168	100%	0.99	87*
Photosystem II D2 protein	psbD	KC954696	Photosynthesis	162	100%	0.98	14*
Photosystem II Q(B) protein	psbA	KC954695	Photosynthesis	137	92%	0.99	14*
Photosystem II 22 kDa protein	PSBS	GO346095.1	Photosynthesis	158	100%	0.99	14*
Photosystem I reaction center subunit IX	PSAJ	GO346974.1	Photosynthesis	160	98%	0.99	14*
Photosystem I reaction center subunit V	PSAG	GO348645.1	Photosynthesis	187	100%	0.99	14*
Chlorophyll *a/b* binding protein 6A	CAB-6A	GO346691.1	Photosynthesis	154	96%	0.99	14*
Chlorophyll *a/b* binding protein 4	LHCA4	GO347781.1	Photosynthesis	200	100%	0.98	14*
Chlorophyll *a/b* binding protein CP29.2	LHCB4.2	GO346860.1	Photosynthesis	195	100%	0.98	14*
Chlorophyll *a/b* binding protein 151	CAB-151	GO347467.1	Photosynthesis	199	93%	0.99	14*
Ferredoxin-1	SEND33	GO348399.1	Electron transport	187	100%	0.98	14*
Ribulose-bisphosphate carboxylase small chain 5B	SSU5B	GO346679.1	Carbon dioxide fixation	169	100%	0.99	14*
Zeaxanthin epoxidase	ZEP	GO348250.1	Xanthophyll cycle	197	100%	0.96	14*
Ubiquinol-cytochrome c reductase iron-sulfur subunit	FES1	GO347392.1	Ubiquinol-cytochrome c reductase complex	196	100%	0.99	F: GGTGATCCAAGCAAGAGAGC
							R: CCACGCCACTTGACTGTCA
Cytochrome c oxidase subunit 5B	COX5B	KC954698	Mitochondrial electron transport chain	181	100%	0.98	F: ACGAGCGGGAGGAGATTG
							R: CAGCCAAAACCAAACAACATC
Alternative oxidase 1A	AOX1A	KC954697	Mitochondrial electron transport chain	116	100%	0.99	F: TGCTGCATTGCAAGTCTCTAC
							R: GTTGTGACACCTCCATGAAGGTC
Malate dehydrogenase	CMDH	GO348392.1	Tricarboxylic acid cycle	235	100%	0.99	F: CCTCATCCTCTCGTCTCCTG
							R: GAGGAAGAGCAGCATCAACC

Gene names are given according to Swiss-Prot best scoring hits. *Reference number.
