# Supplementary material for: Depth-specific fluctuations of gene expression and protein abundance modulate the photophysiology in the seagrass Posidonia oceanica
Source: Sci Rep. 2017 Feb 17;7:42890. doi: 10.1038/srep42890 (PMC5314359; doi:10.1038/srep42890)
Supplement: Supplementary Material [file srep42890-s1.pdf]

DEPTH-SPECIFIC FLUCTUATIONS OF GENE EXPRESSION AND PROTEIN ABUNDANCE MODULATE THE PHOTOPHYSIOLOGY IN THE SEAGRASS POSIDONIA OCEANICA

Gabriele Procaccini[1], Miriam Ruocco[1], Lázaro Marín-Guirao[1], Emanuela Dattolo[1], Christophe Brunet[1],Daniela D'Esposito[1], Chiara Lauritano[1], Silvia Mazzuca[2], Ilia Anna Serra[2], Letizia Bernardo[2],Amalia Piro[2], Sven Beer[3], Mats Björk[4], Martin Gullström[4], Pimchanok Buapet[4,9], Lina M. Rasmusson[4], Paulo Felisberto[5], Sylvie Gobert[6], John W. Runcie[7], João Silva[8], Irene Olivé[8],Monya M. Costa[8], Isabel Barrote[8],Rui Santos[8]

Table S1. Results of the ANOVA analyses for testing for the effects of depth and sampling time on parameters derived from the chlorophyll *a* fluorescence measures (quantum yield of PSII, non-photochemical quenching and electron transport rate), photoprotective pigments (violaxanthin, antheraxanthin, zeaxanthin and xanthophylls de-epoxidation rate), and leaf respiration along the daily cycle here studied.

ANOVA analyses			
Effect	df	F	p
One-way repeated measures ANOVA			
Chlorophyll*a*fluorescencevariables			
Quantum yield of PSII (ΔF/Fm')			
Depth	1	0.35	n.s.
Time	5	268	***
D x T	5	4.43	**
Non-photochemicalquencing (NPQ)			
Depth	1	34.5	**
Time	5	81.2	***
D x T	5	1.06	n.s.
Electron transport rate (ETR)			
Depth	1	184.9	***
Time	3	26.9	*
D x T	3	12.9	*
Two-way factorial ANOVA			
Pigments			
Violaxanthin (V)			
Depth	1	0.915	n.s.
Time	1	1.106	n.s.
D x T	1	0.390	n.s.
Antheraxanthin (A)			
Depth	1	0.00497	n.s.
Time	1	1.363	n.s.
D x T	1	0.888	n.s.
Zeaxanthin (Z)			
Depth	1	2.281	n.s.
Time	1	1.684	n.s.
D x T	1	0.933	n.s.
De-epoxidation ratio (DR)			
Depth	1	8.033	**
Time	1	0.569	n.s.
D x T	1	1.016	n.s.
Leafrespiration			
Respiratoryrates (Rd)			
Depth	1	15.8	***
Time	5	3.19	*
D x T	5	1.68	n.s.

Table S2. Results of Person correlation analyses to assess the direct effects of irradiance (i.e. PAR levels) on *P. oceanica* responses measured at different plant levels (i.e. mRNAs, proteins, respiration and photochemistry).

Correlation analyses with PAR levels									
	5 m			20 m			5 m + 20 m		
Variables	N	r	p	N	r	p	N	r	p
Photosyntheticparameters									
Quantum yield of PSII (ΔF/Fm')	6	-0.97	**0.002**	6	-0.96	**0.003**	12	-0.75	**0.005**
Non-photochemical quenching (NPQ)	6	0.99	**0.0001**	6	0.98	**0.001**	12	0.66	**0.019**
Electron transport rate (ETR)	6	0.79	0.061	6	0.86	**0.0028**	12	0.84	**0.0001**
Photosynthesis-relatedgenes									
PSAJ	6	0.63	0.178	6	0.87	**0.024**	12	0.55	0.063
PSAG	6	0.33	0.52	6	0.68	0.139	12	0.31	0.323
psbA	6	0.88	0.057	6	0.72	0.104	12	0.65	**0.022**
psbD	6	0.38	0.46	6	0.6	0.21	12	0.34	0.285
PSBS	6	0.01	**0.01**	6	0.85	**0.032**	12	0.77	**0.003**
CAB-6A	6	0.39	0.439	6	0.57	0.24	12	0.27	0.392
LHCA4	6	0.91	**0.013**	6	0.74	0.095	12	0.59	**0.043**
CAB-151	6	0.26	0.619	6	0.52	0.29	12	0.22	0.478
LHCB4.2	6	0.27	0.598	6	0.71	0.11	12	0.28	0.371
SEND33	6	0.53	0.283	6	0.78	0.065	12	0.5	0.1
SSU5B	6	0.52	0.286	6	0.44	0.381	12	0.25	0.441
ZEP	6	0.584	0.584	6	0.27	0.604	12	0.21	0.512
Leafrespiration									
Respiratoryrates (Rd)	6	0.37	0.467	6	0.86	**0.034**	12	-0.02	0.958
Respiration-relatedgenes									
CMDH	6	0.5	0.307	6	0.85	**0.032**	12	0.34	0.286
AOX 1A	6	0.89	**0.018**	6	-0.31	0.55	12	0.57	0.054
COX5B	6	0.23	0.665	6	0.68	0.14	12	0.16	0.617
FES1	6	0.62	0.188	6	0.84	**0.035**	12	0.36	0.245
Proteins									
Photosystem II D2 protein	5	0.66	0.222	5	0.98	**0.004**	10	0.73	**0.015**

Significant correlations are indicated in bold

Table S3. Relative expression report for RT-qPCR experiments obtained with REST 2009 [100]. Expression values (with Std. Error and 95% C.I.) and *P(H1)* (Probability of alternate hypothesis that difference between sample and control groups is due only to chance) are reported for each gene and sampling time. Relative quantification of mRNA levels in shallow plants (-5 m) was calculated using plants collected in the deep meadow portion (-20 m) as control condition.

Gene	*PSAJ*	*PSAG*	*psbA*	*psbD*	*PSBS*	*CAB6A*	*LHCA4*	*CAB151*	*LHCB4.2*	*SEND33*	*SSU5B*	*ZEP*	*CMDH*	*AOX1A*	*COX5B*	*FES1*
**06:00**																
Expression	2.060	3.485	8.486	4.964	4.139	1.519	1.361	3.774	3.939	4.065	3.414	2.798	3.398	0.397	1.948	5.777
Std. Error	1.546-2.685	2.392-5.409	3.400-19.627	3.571-6.838	1.283-13.709	0.700 - 2.989	0.657 - 3.325	1.989 - 6.308	2.081 - 7.038	3.278-5.107	2.594 - 4.377	2.045-4.336	1.869 - 4.842	0.153 - 0.864	1.485 - 2.669	3.963-8.270
95% C.I.	1.332-3.308	1.786-6.734	1.941-24.725	2.797-8.106	0.762-32.862	0.467 - 6.076	0.414 - 4.773	1.595 - 11.647	1.470 - 10.848	3.074-5.266	2.456 - 5.409	1.402-4.948	1.756 - 5.237	0.123 - 1.415	1.432 - 3.171	3.689-8.468
P(H1)	0.029	0.100	0.029	0.029	0.154	0.421	0.528	0.029	0.081	0.029	0.029	0.067	0.000	0.187	0.000	0.000
Result	UP		UP	UP				UP		UP	UP		UP		UP	UP
**09:00**																
Expression	1.954	2.346	8.98	7.183	12.241	3.578	2.507	2.957	6.031	3.264	1.596	3.999	4.556	0.474	2.074	7.718
Std. Error	1.132-3.332	0.859-10.766	4.343-14.316	4.599-10.696	6.623-26.355	1.018 - 7.508	0.684 - 6.347	0.890 - 8.953	1.333 - 21.060	1.250-9.425	0.517 - 3.823	2.082-7.319	2.456 - 10.449	0.265 - 1.193	1.717 - 2.410	4.872-12.465
95% C.I.	0.876-4.804	0.564-12.945	4.212-32.629	3.711-15.325	4.053-31.581	0.859 - 8.801	0.585 - 8.746	0.808 - 10.324	1.167 - 29.504	0.825-14.807	0.445 - 5.058	1.598-8.219	1.879 - 14.445	0.232 - 1.333	1.564 - 2.635	4.606-16.799
P(H1)	0.102	0.470	0.000	0.048	0.036	0.222	0.290	0.290	0.000	0.116	0.433	0.072	0.000	0.236	0.053	0.029
Result			UP	UP	UP				UP				UP			UP
**12:00**																
Expression	2.121	2.896	9.017	3.089	9.255	0.868	1.373	1.943	3.013	1.736	1.658	1.849	1.342	4.329	1.459	2.323
Std. Error	1.356-4.071	1.633-5.425	4.785-19.099	1.764-5.559	2.263-29.796	0.420 - 2.049	0.691 - 2.589	1.032 - 3.676	1.391 - 7.576	0.818-5.079	0.898 - 3.974	0.994-3.887	0.670 - 2.865	3.563 - 5.407	0.879 - 2.266	1.606-3.629

95% C.I.		0.870-4.773	1.208-7.389	2.806-20.460	1.228-6.859	1.292-36.765	0.282 - 2.968	0.542 - 4.211	0.768 - 5.577	1.069 - 11.140	0.369-6.470	0.520 - 4.997	0.752-5.848	0.466 - 4.166	3.427 - 5.942	0.694 - 2.471	1.113-4.335
P(H1)		0.123	0.000	0.051	0.073	0.038	0.727	0.524	0.238	0.038	0.437	0.470	0.219	0.472	0.040	0.297	0.090
Result			UP			UP				UP					UP		
	**15:00**																
Expression		1.275	0.57	2.94	1.952	7.892	0.295	1.234	0.595	0.675	5.585	1.06	2.552	2.152	7.615	0.477	2.379
Std. Error		0.976-1.557	0.342-0.908	1.759-5.261	0.390-9.167	2.217-28.991	0.239 - 0.369	0.654 - 3.258	0.197 - 1.392	0.099 - 2.769	1.839-18.371	0.619 - 1.795	0.519-6.121	1.327 - 3.015	1.640 - 22.157	0.355 - 0.698	1.555-3.159
95% C.I.		0.921-1.606	0.278-1.334	1.279-6.601	0.253-15.331	1.039-57.896	0.199 - 0.432	0.441 - 3.833	0.166 - 1.859	0.066 - 3.593	0.802-25.640	0.500 - 2.169	0.453-7.226	1.282 - 3.101	1.096 - 38.004	0.329 - 0.774	1.389-3.311
P(H1)		0.362	0.105	0.065	0.541	0.013	0.000	0.666	0.508	0.846	0.121	0.793	0.29	0.000	0.000	0.100	0.061
Result						UP	DOWN							UP	UP		
	**18:30**																
Expression		1.325	2.039	2.914	1.05	6.405	0.366	0.944	0.853	1.823	1.694	0.453	1.136	1.68	1.434	1.682	3.119
Std. Error		0.965-1.838	1.321-2.804	1.003-8.221	0.448-2.328	1.946-14.769	0.227 - 0.568	0.433 - 1.677	0.568 - 1.306	0.916 - 3.224	1.284-2.503	0.340 - 0.588	0.828-1.442	1.393 - 2.117	0.806 - 2.913	1.285 - 2.296	2.502-3.620
95% C.I.		0.872-2.200	1.218-2.997	0.838-18.902	0.384-4.600	1.366-25.762	0.166 - 0.836	0.397 - 2.056	0.429 - 1.582	0.692 - 4.378	1.111-2.748	0.291 - 0.670	0.723-1.852	1.294 - 2.256	0.446 - 3.718	1.055 - 2.481	2.418-4.589
P(H1)		0.247	0.000	0.214	0.953	0.053	0.063	0.764	0.490	0.207	0.037	0.000	0.395	0.053	0.468	0.000	0.022
Result			UP								UP	DOWN				UP	UP
	**00:00**																
Expression		2.103	2.967	3.252	2.167	7.183	3.23	3.121	2.224	3.804	5.487	4.851	1.856	2.96	1	2.047	3.523
Std. Error		1.503-3.302	1.611-6.269	2.341-4.475	1.523-3.109	4.449-9.810	1.357 - 10.590	1.439 - 12.259	1.036 - 6.160	1.939 - 11.384	2.334-16.455	4.063 - 5.566	1.391-2.673	2.155 - 4.234	0.847 - 1.259	1.590 - 2.924	2.656-5.066

95% C.I.	1.382-3.571	1.376-7.015	1.817-5.505	1.237-3.860	4.111-10.460	0.900 - 14.952	0.945 - 14.190	0.726 - 7.978	1.224 - 13.888	1.658-20.552	3.749 - 6.435	1.077-3.092	1.604 - 5.078	0.711 - 1.327	1.219 - 3.195	1.929-5.976
P(H1)	0.097	0.08	0.081	0.063	0.063	0.134	0.168	0.270	0.041	0.034	0.063	0.019	0.044	0.936	0.036	0.036
Result									UP	UP		UP	UP		UP	UP

Table S4. Accession, description and peptide sequences of proteins that showed at least fivefold changes in their abundance along the daily cycle among plants at two depths. The accessions in bold are from the Illumina dataset. [a]Accession in customized database containing a collection of protein from multiple databases (see [96]) and from the transcriptome of the NGS Illumina dataset (unpublished data). [b]Description based on homology to a sequence in the NCBI non-redundant protein database (see Supplementary Table S5). [c]Mass/charge. [d]Charge of precursor ion.

Accession[a]	Mass (D)	Protein Coverage	Description[b]	Detected peptide sequence	m/z[c]	Z[d]	Score
**gb|GEMD01072936.1|**	25814.97	9.70	Photosystem I reaction center subunit III	EIIIDVPLASK	599.3583	2	31.48
				YAPDSAPALAIK	608.8325	2	26.32
**gb|GEMD01061636.1|**	38906.55	3.12	Photosystem II D1 protein	VINTWADIINR	657.8621	2	48.97
**gb|GEMD01020288.1|**	39621.54	6.23	Photosystem II D2 protein	AAEDPEFETFYTK	774.3489	2	75.71
				NILLNEGIR	521.3065	2	24.45
**gb|GEMD01034542.1|**	34789.44	6.06	Oxygen-evolving enhancer protein 1	VPFLFTIK	482.7972	2	41.03
				ENIKDNTSSTGK	647.3179	2	40.39
**gb|GEMD01035997.1|**	27978.02	12.12	Chlorophyll *a/b* binding protein of LHCII type 1-like	AVPGSPWYGPDR	651.3176	2	49.52
				FGEAVWFK	492.2531	2	36.72
				GPLENLADHLAD	632.8123	2	38.17
				FGEAVWFK	983.4985	1	31.16
Pooc_Contig239	28520.54	6.82	Chlorophyll *a/b* binding protein	SIWYGVDRPK	610.825	2	51.13
				FGEAVWFK	492.2531	2	36.72
				FGEAVWFK	983.4985	1	31.16
**gb|GEMD01071487.1|**	30852.52	12.46	Chlorophyll *a/b* binding protein CP26	TGALLLDGNTLNYFGK	848.9491	2	68.43
				HLSDPFGNNLLTVISSSAER	719.7031	3	47.2
**gb|GEMD01072937.1|**	31510.85	12.15	Chlorophyll *a/b* binding protein CP29.1	STPFQPYTEVFGLQR	885.4467	2	70.7
				LYPGGSFFDPLGLAADPEKK	708.0337	3	37.04
**gb|GEMD01065164.1|**	55393.39	4.73	ATP synthase CF1 alpha subunit, chloroplast	TFTEEAEALLK	626.3273	2	66.63
				IAQIPVSEAYLGR	708.8962	2	57.93
**gb|GEMD01072940.1|**	53986.37	27.37	Ribulose-bisphosphate carboxylase large subunit	FEFKPVDTI	548.2899	2	22.54
				EIKFEFKPVDTI	733.4008	2	40.66
				DLASEGNEIIR	608.8123	2	44.65
				LTYYTPEYETK	704.3378	2	45.34
				TFQGPPHGIQVER	733.3812	2	63.07
				SQAETGEIK	481.7434	2	40.92
				TFQGPPHGIQVER	489.2567	3	28.27
				DTDILAAFR	511.2695	2	51.05
				GGLDFTKDDENVNSQPFMR	724.0008	3	51.56
				AMHAVIDR	456.7399	2	41.13
				EITLGFVDLLR	638.3692	2	52.25
				IPPAYSK	775.4349	1	28.47
				TFQGPPHGIQVER	489.2567	3	31.68
				DDYIEKDR			

				MSGGDHVHAGTVVGK	527.2462	2	35.98
				MSGGDHVHAGTVVGK	726.3569	2	65.76
				MSGGDHVHA	484.5739	3	28.91
				EITLGFVDLLR	726.3569	2	70.94
				DDYIEKDR	770.7377	3	25.54
				GTVVGK	1021.531	1	38.88
				DTDILAAFR			
tr|Q9ATC1|Q9ATC1_ZANAE	37049.54	3.29	RuBisCO activase	IPLILGIWGGK	583.8687	2	43.04
**gb|GEMD01002107.1|**	49726.40	3.13	Phosphoglyceratekinase, chloroplast	GVSLLLPTDVVIADK	770.4535	2	34.14
**gb|GEMD01018778.1|**	36762.09	11.50	Glyceraldehyde-3-phosphate dehydrogenase	AAIKEESEGK	531.2776	2	52.32
				AAGFNIIPSSTGAAK	702.878	2	42.32
				VPTVDVSVVDLTVR	749.9277	2	73.94
**gb|GEMD01077723.1|**	43245.37	17.33	Glyceraldehyde-3-phosphate dehydrogenase A	AAALNIVPTSTGAAK	692.8936	2	36.45
				VVDLADIVANNWK	728.8936	2	68.87
				ENSPLEVIAINDTGGVK	878.4601	2	56.95
				YDSTLGIFDADVK	722.3539	2	70.02
				AVGNNIISVDGK	593.8252	2	42.66
dbj|BAJ87490.1|	16541.32	5.88	Histone H2B.2	LVLPGELAK	470.2976	2	22.84

Table S5. Accession, predicted sequences and NCBI Blastp against non-redundant protein sequences of proteins with fivefold changes in their abundance along the daily cycle. The accessions from the Illumina dataset are marked in bold.

Accession	Predicted protein sequence	Blastp Hit Description	Score	Query cover	Evalue	Ident	Accession
**gb|GEMD01072936.1|**	MASLTTIPSSTLSKRLHHPKLHHSKLK<br>PLTISCSSSITPSPQTPEASNNNNSQLK<br>AFSAAVALSSILLSSPIVPPASADIAGL<br>TPCKESKAFAKREKQSLKKLESSLKK<br>YAPDSAPALAIKASMEKTKRRFDNY<br>GKQGLLCGSDGLPHLIVSGDQRHWG<br>EFITPGILFLYIAGWIGWVGRSYLIAIR<br>DEKKPTMKEIIIDVPLASKLIWRGFIW<br>PVAAYRELINGDLVVDDADVSVT	Photosystem I reaction center subunit III [*Zostera marina*]	371	99%	7e-127	82%	KMZ75766.1
**gb|GEMD01061636.1|**	MTAILERRESTSLWGRFCNWITSTEN<br>RLYIGWFGVLMIPTLLTATSVFIIAFIA<br>APPVDIDGIREPVSGSLLYGNNIISGAII<br>PTSAAIGLHFYPIWEAASVDEWLYNG<br>GPYELIVLHFLLGVACYMGREWELSF<br>RLGMRPWIAVAYSAPVAAASAVFLIY<br>PIGQGSFSDGMPLGISGTFNFMIVFQA<br>EHNILMHPFHMLGVAGVFGGSLFSA<br>MHGSLVTSSLIRETTENESANEGYRF<br>GQEEETYNIVAAHGYFGRLIFQYASF<br>NNSRSLHFFLAAWPVVGIWFTALGIS<br>TMAFNLNGFNFNQSVVDSQGRVINT<br>WADIINRANLGMEVMHERNAHNFPL<br>DLAAVEAPSING	Photosystem II protein D1 [*Typhalatifolia*]	712	100%	0.0	99%	YP_003433955.1
**gb|GEMD01020288.1|**	MTITLGRFTKEENDLFDIMDDWLRRD<br>RFVFVGWSGLLLFPCAYFALGGWFT<br>GTTFVTSWYTHGLASSYLEGCNFLTA<br>AVSTPANSLAHSLLLLWGPEAQGDFT<br>RWCQLGGLWTFVALHGAFGLIGFML<br>RQFELARSVQLRPYNAIAFSGPIAVFV<br>SVFLIYPLGQSGWFFAPSFGVAAIFRFI	Photosystem II D2 protein [*Nymphaea alba*]	715	100%	0.0	99%	YP_053150.1

	LFFQGFHNWTLNPFHMMGVAGVLG AALLCAIHGATVENTLFEDGDGANTF RAFNPTQAEETYSMVTANRFWSQIFG VAFSNKRWLHFFMLFVPVTGLWMSA LGVVGLALNLRAYDFVSQEIRAAEDP EFETFYTKNILLNEGIRAWMAAQDQP HENLIFPEEVLPRGNAL						
**gb|GEMD01034542.1|**	MAASLQATATLMQPTKIGGGFGLQL RSAPQLSKAFGIEGSAGSRLTCSLQTD IKEFASKCADAGKMAGFALATSALL ATGAGAEGVPKRLTFDEVQSQTYME VKGTGTANQCPTIDGGSESFPFKAGK YNMKNLCLEPTSFTVKAEGVAKNSP LEFQKTKLMTRLTYTLSDIEGPFEISS NGKVKFEEKDGIDYAAVTVQLPGGE RVPFLFTIKQLVASGTPDKIEGSFLVPS YRGSSFLDPKGRGGSTGYDNAVALP AGGRGDEEVLAKENIKDNTSSTGKIS FTVTKSKPQTGEVIGVFESIQPSDTDL GAKVPKDVKIQGIWYAQLD	Oxygen-evolving enhancer protein 1, chloroplastic-like [*Solanum tuberosum*]	547	100%	0.0	81%	XP_006338257.1
**gb|GEMD01035997.1|**	MAATMALSSPSLAGKAVKLAPSASE TLGAGRVTMRSTIKSKAVPGSPWYGP DRVKYLGPLSGEAPSYLTGEFPGDYG WDTAGLSADPETFAKNRELEVIHCR WAMLGTLGCVFPELLSRNGVKFGEA VWFKAGSQIFSDGGLDYLGNPSLVH AQSILAIWACQVILMGAVEGYRIAGG PLGEVVDPLYPGGSFDPLGLAEDPEA FAELKVKEIKNGRLAMFSMFGFYVQ AIVTGKGPLENLADHLADPVNNNAW AYATNFVPGK	Chlorophyll *a/b* binding protein of LHCII type 1-like [*Musa acuminata* subsp. malaccensis]	501	100%	5e-177	93%	XP_009389379.1
Pooc_Contig239	MATSAIQQSAFAGQTALKQSNELIRK VGVSEGRITMRRTVKSAPKSIWYGVD RPKYLGPFSEQTPSYLTGEFPGDYGW DTAGLSADPETFAKNRELEVIHARWA MLGTLGCVLPELLAKNGVKFGEAVW FKAGSQIFSEGGLDYLGNPNLVHAQS ILAIWACQVVLMGFIEGYRVGGGPLG EGLDALYPGGSFDPLGLADDPDAFAE	Chlorophyll *a/b* binding protein [*Ziziphusjujuba*]	511	100%	0.0	92%	AHC95482.1

	LKVKEIKNGRLAMFSMFGFYVQAIVT GKGPIENLYDHLADPVANNAWAYAT NFVPGK						
**gb|GEMD01071487.1|**	MASLAAAPASLGMTEMLGSPLQFSG PARTAPVQSGAPSSKIVALFSKKPKRP PAKAKSAAVSPASDELAKWYGPDRR IYLPEGLLDRSEVPEYLNGEVPGDYG YDPFGLSKKPENFAKYQAFELIHARW AMLGAAGFVIPEAFNKFGASCGPEAV WFKTGALLLDGNTLNYFGKNIPINLA IAVIAEVVLVGGAEYYRITNGLDLKD KLHPGGPFDPLGLASDPDQTALLKVK EIKNGRLAMFAMLGFFIQAYVTGEGP VENLSKHLSDPFGNNLLTVISSSAERV PTL	Chlorophyll *a/b* binding protein CP26, chloroplastic-like [*Elaeisguineensis*]	492	96%	1e-172	85%	XP_010910942.1
**gb|GEMD01072937.1|**	MATAAAAATSAFLGSRLPEVSSPANS RFQARFGFGRKKRAPKKPSRASTTNR PLWYPGAKSPEWLDGTLVGDYGFDP FGLGKPAEYLQFDLDSLDQNLAKNN VGDLIGTRFETADVKSTPFQPYTEVF GLQRFRECELIHGRWAMLATLGALA VESLTGVTWQDAGKFELVDGSSYLG QPLPFSISTLIWIEVLVIGYIEFQRNAE LDAEKRLYPGGSFFDPLGLAADPEKK ATLQLAEIKHARLAMVGFLGFAVQA WATGKGPLNNWATHLSDPLHTTIFD TLGLFS	Chlorophyll *a/b* binding protein CP29.1, chloroplastic-like [*Solanum tuberosum*]	484	98%	5e-170	86%	XP_006366348.1
**gb|GEMD01065164.1|**	MVTLRADEISNIIRERIEQYNREVKVV NTGTVLQVGDGIARIHGLDEVMAGE LVEFEEGTVGIALNLESNNVGVVLMG DGLMIQEGSSVKATGRIAQIPVSEAYL GRVINALAKPIDGRGEISSSESRLIESP APGIISRRSVYEPLQTGLIAIDSMIPIGR GQRELIIGDRQTGKTAVATDTILNQK GQNVICVYVAIGQKASSVAQVVTTFQ ERGAMEYTIVVAETADSPATLQYLAP YTGATLSEYFMYCQRHTLIIYDDLSK QAQAYRQMSLLLRRPPGREAYPGDV FYLHSRLLERAAKLSSRLGEGSMTAL PIVETQSGDVSAYIPTNVISITDGQIFL	ATP synthase CF1 alpha subunit, chloroplast [*Colpothrinaxcookii*]	983	99%	0.0	96%	YP_009170162.1

	SGDLFNSGIRPAINVGISVSRVGSAAQ IKAMKQVAGKLKLELAQFAELEAFA QFASDLDKATQNQLARGQRLRELLK QPQSDPLTVGEQIVTIYTGTNGYLDSL EIGQVKKFLVQLRTYLKKNKPEFQEII SSTKTFTEEAEALLKEAIKEQIELFLIQ EQG						
**gb|GEMD01072940.1|**	MSCREGLMLPQTETKASAGFKAGVK DYKLTYYTPEYETKDTDILAAFRVTA QPGVPPEEAGAAVAAESSTGTWTTV WTDGLTSLDRYKGRCYHIEPVVGEE DQFIAYVAYPLDLFEEGSVTNMFTSIV GNVFGFKALRALRLEDLRIPPAYSKT FQGPPHGIQVERDKLNKYGRPLLGCT IKPKLGLSAKNYGRAVYECLRGGLDF TKDDENVNSQPFMRWRDRFLFCAEA IYKSQAETGEIKGHYLNATAATCEEM etLKRAIFARELGVPIVMetHDYLTGGF TANTTLSYYCRDNGLLLHIHRAMHA VIDRQKNHGMHFRVLAKALRMSGG DHVHAGTVVGKLEGEREITLGFVDLL RDDYIEKDRSRGIFFTQDWVSMPGVF PVASGGIHVWHMPALTEIFGDDSVLQ FGGGTLGHPWGNAPGAVANRVALE ACVKARNEGRDLASEGNEIIREASKW SPELAAACEVWKEIKFEFKPVDTI	Ribulose-1,5-bisphosphate carboxylase/oxygenase large subunit [*Asparagus officinalis*]	941	100%	0.0	95%	AFA27665.1
tr|Q9ATC1|Q9ATC1_ZANAE	THNPVLSSYEYISTGLRSFNLDNTVN GLYIAPAFMDKLVVHITKNFMNLPNI KIPLILGIWGGKGQGKSFQCELVFAK MGINPIMMSAGELESGNAGEPAKLIR QRYREAADIIRKGKMCCLFINDLDAG AGRMGGTTQYTVNNQMVNATLMNI ADNPTNVQLPGMYNKQENPRVPIIVT GNDFSTLYAPLIRDGRMEKFYWAPTR DDRVGVCTGIFRSDNVPKEDVIKLVD TFPGQSIDFFGALRARVYDDEVRKWI AEIGVDGVGKRLVNSLEGPPTFAQPK MTLDKLLEYGNMLVQEQENVKRVQ LADKYLSEAALGDANQDAIKTGSFY G	RuBisCO activase [*Zantedeschiaaethiopica*]	695	100%	0.0	100%	AAK25801.1

**gb|GEMD01002107.1|**	MAAAAASTSFSLLSSTRRATAAAAA<br>VPRGITLKATPRRLGFSGAAADSVLA<br>LHVAERIRAVAGRSGRGPRGVAAMS<br>KRSVGDLSEAELKGKKVFVRADLNV<br>PLDDGQNITDDTRIRAAVPTIKHLIGK<br>GAKVILTSHLGRPKGVTPKFSLAPLVP<br>RLGELLGVKVEKADDCVGPEVQKVV<br>DALPEGGVLLLENVRFHKEEEKNDPE<br>FAQKLAALADLYVNDAFGTAHRAHA<br>STEGVTKYLKPSVSGFLLQKELDYLV<br>GAVSSPKRPFAAIVGGSKVSSKIGVIE<br>SLLENVNILLLGGGMIFTFYKAQGLST<br>GSSLVEEDKLELAKSLMAKAKAKGV<br>SLLLPTDVVIADKFAPDANCQTVPAS<br>AIPDGWMGLDIGPDSVKTFNEALETT<br>QTVIWNGPMGVFEFDKFAVGTESVA<br>KKLAELSGKGVTTIIGGGDSVAAVEK<br>VGVADLMSHISTGGGASLELLEGKPL<br>PGVLALDEAVTVAA	Phosphoglyceratekinase, chloroplast<br>[*Zostera marina*]	816	99%	0.0	84%	KMZ64101.1
**gb|GEMD01018778.1|**	MAGGKTKIGINGFGRIGRLVARVALQ<br>RDDVELVAVNDPFITTDYMTYMFKY<br>DTVHGQWKHHDIKVKDSKTLLFGEK<br>TVTVFGIRNPEEIPWGETGAEYVVEST<br>GVFTDKAKAAAHLQGGAKKVVISAP<br>SKDAPMFVMGVNEHEYKCDIDIVSN<br>ASCTTNCLAPLAKVINDKFGIVEGLM<br>TTVHSVTATQKTVDGPSSKDWRGGR<br>AAGFNIIPSSTGAAKAVGKVLPALNG<br>KLTGMAFRVPTVDVSVVDLTVRLKK<br>SATYEQIKAAIKEESEGKMKGILGYV<br>EEDLVSTDFVGDCRSSIFDAKAGIALS<br>ENFVKLVSWYDNEWGYSSRVVDLIV<br>HMSETH	Glyceraldehyde-3-phosphate dehydrogenase<br>[*Ananas comosus*]	633	98%	0.0	91%	ADQ43814.1
**gb|GEMD01077723.1|**	MASSTLFSAATSSLQAVNGKGFSDFS<br>GLRSSAQLPVAKKLHSDDFFSVISFQT<br>AAVRGDGYKKGVVEAKVKVAINGF<br>GRIGRNFLRCWHGRENSPLEVIAINDT<br>GGVKQASHLLKYDSTLGIFDADVKA<br>VGNNIISVDGKEVRVVSDRNPANLPW<br>KEMGIDLVIEGTGVFVDKEGAGKHL	Glyceraldehyde-3-phosphate dehydrogenase A, chloroplastic<br>[*Nelumbo nucifera*]	675	100%	0.0	89%	XP_010250427.<br>1

	QAGAKKVLITAPGKGDIPTYVVGVN ADAYNPDEPIISNASCTTNCLAPFVKV LDQKFGIIKGTMTTTHSYTGDQRLLD ASHRDLRRARAAALNIVPTSTGAAKA VALVLPGLKGKLNGIALRVPTPNVSV VDLVVQVSKKTFAEEVNAAFRESAD KELKDILVVCDEPLVSVDFRCSDVSS TVDSSLTMVMGDDMVKVIAWYDNE WGYSQRVVDLADIVANNWK						
dbj|BAJ87490.1|	MAPKADKKPAAENKVEKAAEKTPA GKKPKAEKRLPAGKTASKEAGGEAK TRGRKKGSKAKKSVETYKIYIFKVLK QVHPDIGISSKAMSIMNSFINDIFEKLA GESAKLARYNKKPTITSREIQTSVRLV LPGELAKHAVSEGTKAVTKFTSS	Histone H2B.2 [*Triticumaestivum*]	286	96%	4e-96	98%	P05621.2

Table S6. Results of the ANOVA analysis for testing for the effects of depth and sampling time on proteinswhich showed fivefold change of variation in the accumulation levels.

Two-way factorial ANOVA				
Effect		df	F	p
	Proteins			
PSI reaction center sub III				
	Depth	1	16.64	***
	Time	4	9.58	***
	D x T	4	1.98	n.s.
Photosystem II D1 protein				
	Depth	1	482.6	***
	Time	4	208.9	***
	D x T	4	77.4	***
Photosystem II D2 protein				
	Depth	1	29.88	***
	Time	4	1.82	n.s.
	D x T	4	1.29	n.s.
Oxygen-evolvingenhancerprotein 1				
	Depth	1	142.3	***
	Time	4	10.85	***
	D x T	4	7.37	***
Chlorophyll a/b binding protein of LHCII type 1				
	Depth	1	16.71	***
	Time	4	0.38	n.s.
	D x T	4	1.07	n.s.
Chlorophyll a/b binding protein				
	Depth	1	12.93	**
	Time	4	5.85	**
	D x T	4	2.81	n.s.
Chlorophyll a/b binding protein CP26				
	Depth	1	27.25	***
	Time	4	9.49	***
	D x T	4	10.97	***
Chlorophyll a/b binding protein CP29.1				
	Depth	1	45.9	***
	Time	4	0.89	n.s.
	D x T	4	7.34	***
ATP synthase CF1 alpha subunit				
	Depth	1	21.8	***
	Time	4	4.31	*
	D x T	4	6.06	**
Ribulose-1,5-Bisphosphate Carboxylase/Oxygenase large subunit				
	Depth	1	32.8	***
	Time	4	6.46	**
	D x T	4	9.57	***
RuBisCOactivase				

	Depth	1	117.3	***
	Time	4	71.67	***
	D x T	4	71.56	***
Phosphoglycerate kinase				
	Depth	1	15.4	***
	Time	4	6.31	**
	D x T	4	1.79	n.s.
Glyceraldehyde-3-phosphate dehydrogenase				
	Depth	1	26.82	***
	Time	4	3.88	*
	D x T	4	2.30	n.s.
Glyceraldehyde-3-phosphate dehydrogenase A				
	Depth	1	19.46	***
	Time	4	6.48	**
	D x T	4	3.94	*
Histone H2B.2				
	Depth	1	15.2	***
	Time	4	9.08	***
	D x T	4	6.9	**
Sum of allproteins				
	Depth	1	317.15	***
	Time	4	37.83	***
	D x T	4	30.02	***

Table S7. Population genetic diversity and differentiation related to depth. N, number of samples; MLG, Multi Locus Genotypes; *R*, (MLG-1)⁄(N-1); A, number of alleles; PA, private alleles; %pol, percentage of polymorphic loci; H_o, observed heterozygosity; uH_e, unbiased expected heterozygosity. *R* has also been calculated for N = 21, the lowest number of individuals analyzed.

	N	MLG	*R*	R_{21}	A	A/locus	PA	% pol.	H_o	uH_e
- 5 m	21	20	0.950	0.950	63	2.86 ±1.61	17	75.86%	0.669 ±0.34	0.451 ±0.19
-20 m	30	24	0.793	0.838	48	2.18 ±1.33	2	65.52%	0.706 ±0.41	0.419 ±0.21

Table S8. F_{IS} values for the shallow and deep sites in the STARESO *P. oceanica* population. In bold F_{IS} significant at 99% confidence interval and P-values of exact test of Hardy-Weinberg equilibrium (asterisks indicate significant departure from Hardy-Weinberg equilibrium, *= *p*<0.05); m = monomorphic locus.

	Shallow		Deep	
	**F_{IS}**	**p**	**F_{IS}**	**p**
Poc-45	-0.900	0.000*	-0.484	0.024*
Po-5	-0.900	0.000*	-1.000	0.000*
Poc-5	m		m	
Poc-35	-0.468	0.013*	0.000	1.000
Po-5-49	-0.555	0.007*	-0.917	0.000*
Poc-trn	1.000	0.001*	m	
Poc-26	m		m	
Po-15	-0.495	0.007*	0.785	0.007*
Po-5-10	-0.827	0.000*	0.000	1.000
Po-4-3	-0.583	0.022*	-0.917	0.000*
Po-5-39	-0.027	1.000	-1.000	0.000*
Po-5-40	0.345	0.007*	-0.243	0.038*
Poc-42	-0.691	0.005*	-1.000	0.000*
Pooc-229	0.240	0.353	m	
Pooc-264	m		m	
Pooc-330	-0.056	1.000	m	
Pooc-3	-0.587	0.008*	-1.000	0.000*
Pooc-54	m		m	
Pooc-153	-0.086	1.000	0.000	1.000
Pooc-333	m		m	
Pooc-125	m		m	
Pooc-PC045G11	-0.126	0.187	-0.183	0.003*
Pooc-PC044B02	-0.652	0.005*	-0.913	0.000*
Pooc-214	-0.027	1.000	m	
Pooc-300d	m		m	m
Pooc-PC047G07	-1.000	0.000*	-1.000	0.000*
Pooc-50	-1.000	0.000*	-1.000	0.000*
Pooc-361	-0.572	0.001*	-1.000	0.000*
Pooc-PC003H09	-0.226	0.548	-0.150	1.000
All	-0.473		-0.709	
SD	0.497		0.537	

Table S9. Analysis of Molecular Variance (AMOVA) results showing degrees of freedom (df), sum of squares (SS), mean sum of squares (MS), estimated variance (Est. Var.) and percentage of variation (%).

Level of variation	**df**	**SS**	**MS**	**Est. Var.**	**%**
Among Depths	1	78.122	78.122	3.409	48%
Within Depths	42	157.242	3.744	3.744	52%
Total	43	235.364		7.153	100%
